# Supplementary material for: Controlled formation of three-dimensional cavities during lateral epitaxial growth
Source: Nat Commun. 2024 Mar 13;15:2247. doi: 10.1038/s41467-024-46222-x (PMC10933262; doi:10.1038/s41467-024-46222-x)
Supplement: Supplementary file 1 — Supplementary Information [file 41467_2024_46222_MOESM1_ESM.pdf]

Supplementary Information

Controlled formation of three-dimensional cavities during lateral epitaxial growth

Yiwen Zhang[1,4], Baoming Wang[2,4], Changxu Miao[3,4], Haozhi Chai[1], Wei Hong[3,*], Frances M. Ross[2,*], Rui-Tao Wen[1,*]

[1]Department of Materials Science and Engineering, Southern University of Science and Technology, Shenzhen, Guangdong 518055, China

[2]Department of Materials Science and Engineering, Massachusetts Institute of Technology, Cambridge, MA, 02139, USA

[3]Shenzhen Key Laboratory of Soft Mechanics and Smart Manufacturing, Department of Mechanics and Aerospace Engineering, Southern University of Science and Technology, Shenzhen, Guangdong 518055, China

[4]These authors contributed equally to this work.

*Corresponding author. Email: hongw@sustech.edu.cn, fmross@mit.edu, wenrt@sustech.edu.cn

The PDF file includes:

Supplementary Notes

Figs. S1 to S19

Table S1

References

Supplementary Notes

Supplementary Note 1: Thermodynamics and kinetics

Fig. S9 illustrates the main idea of the phase-field model developed to understand the underlying mechanisms of the ELO and coalescence phenomenon. Two concurrent processes are modeled: the gas-solid phase transition of Ge and the transport of Ge atoms. Phase-field models are commonly used in simulating phase transition processes and the corresponding interfacial evolution. A continuous and conserved phase field is introduced to capture kinetics of quasi-immobile solidified Ge atoms, oversaturated gaseous Ge and Ge atoms on interfacial layers.

A continuous order parameter, $C(\mathbf{x}, t)$, is introduced as a function of the spatial coordinates $\mathbf{x}$ and time t. We select the normalized concentration of Ge as the order parameter, $C(\mathbf{x}, t) = (\rho(\mathbf{x}, t) - \rho^{\mathrm{G}})/(\rho^{\mathrm{S}} - \rho^{\mathrm{G}})$, where ρ is the local Ge concentration, and ρ^{S} and ρ^{G} are the equilibrium concentrations of the coexisting solid and gas phases, respectively. The order parameter varies continuously but abruptly between $C = 0$ in the saturated vapor and $C = 1$ in the solid phase; the narrow transition zone can be regarded as a representation of the surface with a layer of irregular atomic packing at the gas/solid interface.

Following the common practice of the Cahn-Hilliard models[1], we write the free energy density into two terms. A simple double-well function $\Phi_{\mathrm{DW}}(C) = \eta C^2(C-1)^2$, with minima at $C = 1$ and 0, is introduced to separate the system into coexisting solid and gas phases. The parameter η characterizes the energy barrier between the two phases. The second contribution, a gradient term, $\Phi_{\mathrm{G}} = \frac{\gamma}{2}|\nabla C|^2$ is always positive. A comparison between the two energy contributions leads to a length scale $L_0 = \sqrt{\gamma/2\eta}$, which characterizes the equilibrium thickness of the solid-gas interface. In summary

$$\Phi(C, \nabla C) = \Phi_{\mathrm{DW}} + \Phi_{\mathrm{G}} = \eta C^2(C-1)^2 + \frac{\gamma}{2}\frac{\partial C}{\partial x_i}\frac{\partial C}{\partial x_i}, \qquad \text{(S1)}$$

with repeated index representing a summation.

When the domain is connected to a reservoir of Ge atoms with chemical potential μ, we let N be the number of Ge inserted into the domain from the reservoir. The total potential energy of the system, including the reservoir,

$$F = \int_{\Omega} \Phi \, \mathrm{d}V - \mu N, \tag{S2}$$

reaches a stationary point, $\delta F = 0$. The integration in Eq. (2) is carried out over the entire computational domain Ω. Conservation of Ge atoms requires that $N = \int_{\Omega} C dV$. Through variational calculations and integration by parts, we obtain the Euler equation:

$$\mu = -\gamma \frac{\partial^2 C}{\partial x_i \partial x_i} + 2\eta C(C-1)(2C-1), \tag{S3}$$

which can be solved for the equilibrium phase field $C(\mathbf{x})$, when proper boundary conditions are imposed.

Now let us consider a general non-equilibrium process. We still assume local equilibrium, so that the inhomogeneous field of chemical potential $\mu(\mathbf{x}, t)$ is still given by Eq. (S3). Writing the chemical potential in the form of Eq. (S3) also implies the choice of taking the equilibrium coexisting states as the reference, in which $\mu = 0$. The first term on the right-hand-side of Eq. (S3) accounts for the mean-curvature contribution on an interface[2, 3], with a non-vanishing gradient of C. The second term corresponds to the chemical potential in the bulk, far from the interface. The two states, $C = 1$ and $C = 0$, share the same chemical potential, corresponding to the coexisting phases.

To model the migration of Ge atoms, we adopt linear kinetics and assume that the diffusion flux is related to the gradient of chemical potential through:

$$j_i = -M_{ik} \frac{\partial \mu}{\partial x_k}. \tag{S4}$$

The positive-definite mobility tensor $\mathbf{M}$ captures both the differential diffusivities between the gas and solid phases, and the contribution from interface diffusion. Here, we write the mobility in the following anisotropic form:

$$M_{ik} = M_{\mathrm{B}} \delta_{ik} + M_{\mathrm{S}} \left(\frac{\partial C}{\partial x_j} \frac{\partial C}{\partial x_j} \delta_{ik} - \frac{\partial C}{\partial x_i} \frac{\partial C}{\partial x_k} \right). \tag{S5}$$

The first term on the right-hand-side captures bulk diffusion, with the phase-dependent isotropic mobility $M_{\mathrm{B}}(C) = m^{\mathrm{S}} + (1-C)^5 m^{\mathrm{G}}$ interpolated between $M_{\mathrm{B}}(0) = m^{\mathrm{G}}$ in gas phase and a much lower level $M_{\mathrm{B}}(1) = m^{\mathrm{S}}$ in solid phase. The second term represents the surface diffusion. The resulting transversely isotropic mobility tensor has a principal value M_{B} in the through-

thickness direction of the interface (along ∇C) and two equal principal values $M_{\mathrm{B}} + |\nabla C|^2 M_{\mathrm{S}}$ along the other two directions perpendicular to the interface normal. Note that $|\nabla C|$ takes non-zero values only within the transition zone between two phases, where the surface diffusion contribution is effective. It is also possible to extend the current model to include anisotropic diffusion (e.g. that due to Ehrlich-Schwoebel barriers[4]), by introducing orientation dependence in the already anisotropic mobility tensor $\mathbf{M}$ in Eq. (S4). Anisotropic diffusion changes the kinetics by directing adatom flux along preferred directions, but does not alter the thermodynamics. Therefore, it may delay some phenomena (e.g. the coalescence), but cannot prevent them from happening.

Finally, the conservation of Ge atoms dictates that

$$\frac{\partial C}{\partial t} = -\frac{\partial j_i}{\partial x_i}, \tag{S6}$$

in the bulk, and

$$j_i n_i = I \tag{S7}$$

on a boundary of unit normal vector $\mathbf{n}$ and known outward flux of Ge, I. Substitution of Eqs. (S3) and (S5) into (S4), and then into (S6), we arrive at a partial differential equation for the evolution of the phase field $C(\mathbf{x}, t)$, which is then written into a weak form, and solved numerically by using a finite element method through the commercial package COMSOL Multiphysics.

Supplementary Note 2: Contact angle

Ge crystals share the same lattice structure as Si, but differs from that of SiO_2. The difference in coherency results in the preferential growth of Ge on Si surface, and the different wettability of Ge on Si and SiO_2. To account for the different surface energy, an additional boundary condition should be imposed on the SiO_2 surface[5]. To derive a consistent boundary condition, let us consider the simplest one-dimensional case, with a flat and infinitely large planar phase boundary dividing the entire space into two.

Let ξ be the one-dimensional coordinate, its axis normal to the solid-gas interface, pointing from the gas phase to the solid phase. The coexisting state of the two phases is still taken as the reference for chemical potential, $\mu = 0$. The equilibrium condition, Eq. (S3), now becomes

$$-\gamma\frac{\mathrm{d}^2C}{\mathrm{d}\xi^2}+\frac{\mathrm{d}\Phi_{\mathrm{DW}}}{\mathrm{d}C}=0. \tag{S8}$$

Multiplying both sides of Eq. (S8) by $\mathrm{d}C/\mathrm{d}\xi$, we arrive at

$$\frac{\mathrm{d}\Phi_{\mathrm{DW}}}{\mathrm{d}\xi}=\frac{\gamma}{2}\frac{\mathrm{d}}{\mathrm{d}\xi}\left(\frac{\mathrm{d}C}{\mathrm{d}\xi}\right)^2. \tag{S9}$$

Integration of both sides of Eq. (S9) from deep inside the bulk gas phase, where $C=0$, $\mathrm{d}C/\mathrm{d}\xi=0$, and $\Phi_{\mathrm{DW}}=0$, to a point near the interface leads to

$$\left(\frac{\mathrm{d}C}{\mathrm{d}\xi}\right)^2=\frac{2\Phi_{\mathrm{DW}}}{\gamma}. \tag{S10}$$

Thus, the slope profile of the equilibrium phase field near an interface takes the form

$$\frac{\mathrm{d}C}{\mathrm{d}\xi}=\sqrt{\frac{2\Phi_{\mathrm{DW}}}{\gamma}}. \tag{S11}$$

Now let us consider the case of a Ge atom deposited on a SiO_2 surface. Near the gas-solid interface, when the curvature effect to the local phase-field profile is negligible, we may assume the local phase-field profile to be close to that of the ideal one-dimensional case, as given by Eq. (S11). As sketched in **Fig. S12a**, in equilibrium, the gas-solid interface of Ge forms a specific angle α with the SiO_2 surface, known as the static contact angle. Thus, the projection of the phase-field gradient ∇C onto the normal direction of the SiO_2 surface can be evaluated as

$$n_i\frac{\partial C}{\partial x_i}=\frac{\mathrm{d}C}{\mathrm{d}\xi}\cos\alpha=\sqrt{\frac{2\Phi_{\mathrm{DW}}}{\gamma}}\cos\alpha=\frac{1}{L_0}C(1-C)\cos\alpha. \tag{S12}$$

We therefore take Eq. (S12) as the boundary condition for a SiO_2 surface.

To validate the effectiveness of the boundary condition, a series of numerical experiments have been carried out. With different values of α taken and boundary condition as Eq. (S12) imposed, finite-element calculations all show that the corresponding contact angles are achieved when the system equilibrates after sufficient time, as shown by **Fig. S12b**. In the current study, the value $\alpha\approx 120°$ is taken from experimental observation.

Supplementary Note 3: Growth front evolution process

To quantify the growth-front evolution process, we first compare the growth front morphology of anisotropic ELO on a square mask, as shown in **Fig. S16(a-c)**. We take Si [110] and [100] as

representative directions for observing the crystal geometry as epi-growth proceeds. During anisotropic growth, no overgrowth was observed at the edge of square mask along Si [110], and the morphology of the upper part of the growth front was defined by {105}, {111} and {113} facets. Along Si [100], where Ge overgrowth started at the four corners of the mask, an obtuse contact angle of 100° was found.

There are three stages of growth front evolution after the anisotropic-isotropic transition, illustrated in **Fig. S17**.

Stage 1: contact angle of the growth front changing from <90° to ~100°, showing a uniform morphology of growth fronts from all directions towards the center of mask. The chemical potential gap between corner and edge vanishes, suggesting a uniform inward growth velocity.

Stage 2: contact angle between Ge growth front and mask is around 100°, suggesting the growth front moves forward with a uniform morphology. This stage only exists when the mask diameter is large enough that the growth fronts proceed independently of each other, as shown in **Fig. S16(d-e)**.

Stage 3: contact angle increases sharply from ~100° to 135°, indicating the front morphology is strongly affected by the negative curvature-induced adatom migration.

Supplementary Note 4: Orientation-dependent surface energy

To arrive at the facetted surface of a single crystal, following Siem and Carter[6], we can replace the interfacial energy coefficient γ with an orientation-dependent parameter in the form of

$$\gamma(\mathbf{n}) = \gamma_0 \left[1 - \sum_{i=1}^{M} \beta^i \left(n_j q_j^i\right)^{w^i} H\left(n_k q_k^i\right) \right]. \tag{S13}$$

where $\mathbf{n} = \nabla C / |\nabla C|$ is the unit normal vector of the solid-gas interface, $\mathbf{q}^i$ is the unit normal vector of one of the M facet surfaces, β^i and w^i are positive parameters controlling the width and depth of the corresponding energy well[6], and $H(x)$ is the Heaviside function. The values of β^i and w^i along particular directions are obtained from refs. (7,8), as listed in Table S1.

Table S1 Facet energy parameters for single-crystal Ge

Facet family	{111}	{001}	{113}	{110}	{313}	{15 3 23}	{21 9 29}
β^i	0.206	0.196	0.179	0.160	0.155	0.153	0.153

w^i	50	50	100	50	50	50	50

Supplementary Note 5: Non-dimensionalization and intrinsic lengths

The time needed for Ge atoms to migrate over the characteristic length of interface thickness, L_0, defines a characteristic time $t_0 = \gamma/2\eta^2 m^{\mathrm{G}}$. We normalize all lengths by L_0, time by t_0, and energy density by η, and represent the dimensionless quantities by hatted symbols corresponding to the original ones. Consequently, the dimensionless free energy density takes the form

$$\hat{\Phi}(C) = C^2(C-1)^2 + \frac{\partial C}{\partial \hat{x}_i}\frac{\partial C}{\partial \hat{x}_i}. \tag{S15}$$

The dimensionless equation for the diffusion of Ge atoms becomes:

$$\frac{\partial C}{\partial \hat{t}} = -\frac{\partial}{\partial \hat{x}_i}\left(-\widehat{M}_{ik}\frac{\partial \hat{\mu}}{\partial \hat{x}_k}\right). \tag{S16}$$

The boundary condition for the contact angle is also written in a dimensionless form

$$n_i \frac{\partial C}{\partial \hat{x}_i} = C(1-C)\cos\alpha\,. \tag{S17}$$

The normalized system has three dimensionless kinetic parameters: $\widehat{m}^{\mathrm{S}} = m^{\mathrm{S}}/m^{\mathrm{G}}$, the mobility of Ge atom diffusion in the bulk solid phase relative to that in the gas phase; $\widehat{M}_S = 2\,M_S\eta/\gamma\, m^{\mathrm{G}}$ representing the mobility of surface diffusion relative to that of the bulk diffusion in the gas phase; and the dimensionless rate of deposition $\hat{I} = I\sqrt{\gamma/2\eta}/\eta m^{\mathrm{G}}$. The diffusion of Ge in the solid phase is deemed as slow and unimportant to the process of interest, thus we select a small enough value $\widehat{m}^{\mathrm{S}} = 1 \times 10^{-6}$. With different values of $\widehat{M}_{\mathrm{S}} = 0,\ 200$ and 400 taken to illustrate their effect, simulations in **Fig. S15** show that growth of Ge is accompanied by the receding of the surrounding surface when surface diffusion is dominant, while no receding is observed when the surface diffusion is reduced. Consequently, $\widehat{M}_{\mathrm{S}} = 400$ is taken to match the observed profile of the growth front.

The simulated domain takes a cubic space as shown in **Fig. S11**. As boundary conditions, the normalized Ge concentration $C = 1$ on the bottom surface, where the dimensionless chemical potential $\hat{\mu} = 0$. All four side walls are impermeable to Ge atoms. Gaseous Ge atoms enter the domain from the top surface at a known flux $\hat{I}$. As initial conditions, $C = 0$ and $\hat{\mu} = 0$ are prescribed anywhere in the domain.

Aside from the characteristic interface thickness L_0, at a deposition rate $\hat{I} = 5 \times 10^{-4}$, the dimensionless characteristic length for the kinetic process is $\hat{L}_s = \frac{L_S}{L_0} = \sqrt[3]{\widehat{M}_S/\hat{I}} \approx 93$. When the characteristic size of the system is smaller than L_s, surface diffusion dominates the Ge migration, and when the scale of the system is larger than L_s, surface diffusion becomes ineffective but influx-induced deposition dominates, leading to a normal growth observed in the early stage of Ge growth on large mask.

Supplementary Figures

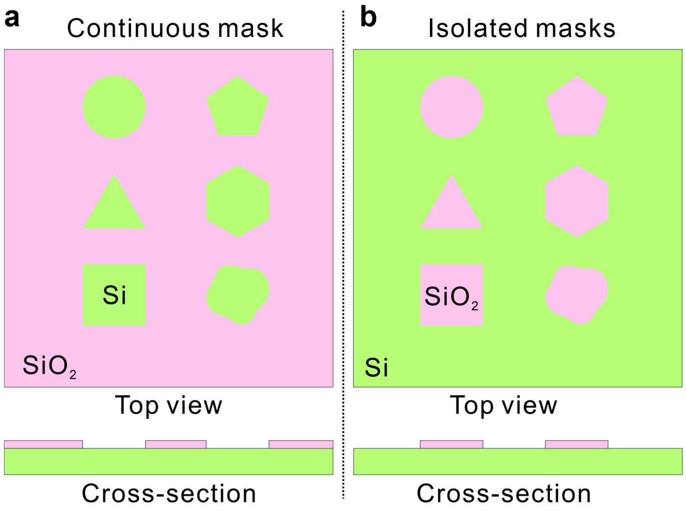

Fig. S1 Illustration of continuous masks and isolated masks. a, isolated growth windows are exposed for epi-growth on continuous mask. **b**, masks are free-standing on continuous Si surface and lateral overgrowth fronts converge over the masks as the epi-growth proceeds.

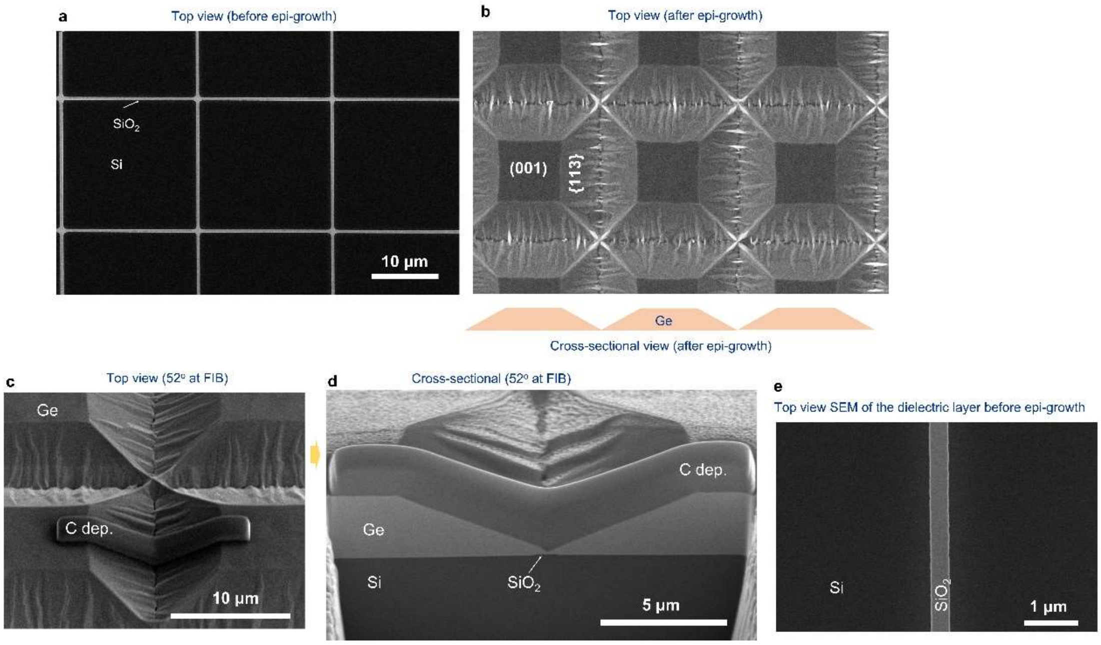

Fig. S2 Low temperature growth of Ge on patterned Si (001). **a**, the mask was in 20×20 μm^2 square shape with oxide width of 1.0 μm. **b**, Top view SEM image of this pattern after epi-growth. The epi-growth Ge was in a trapezoid shape with dominant facets of {311}. Moreover, the profile of the epi-Ge was rather rough due to the low mobility of Ge adatoms during growth. **c**, tilted SEM view of the low temperature epi-Ge after carbon deposition for FIB milling. Ravines can be clearly seen on low-temperature Ge. **d,** tilted view of the oxide stripe, showing oxide is covered by Ge without forming tunnel or cavity. **e**, lithography defined oxide margins, imperfections can be clearly observed which led to the Ge ravines after growth due to low mobility.

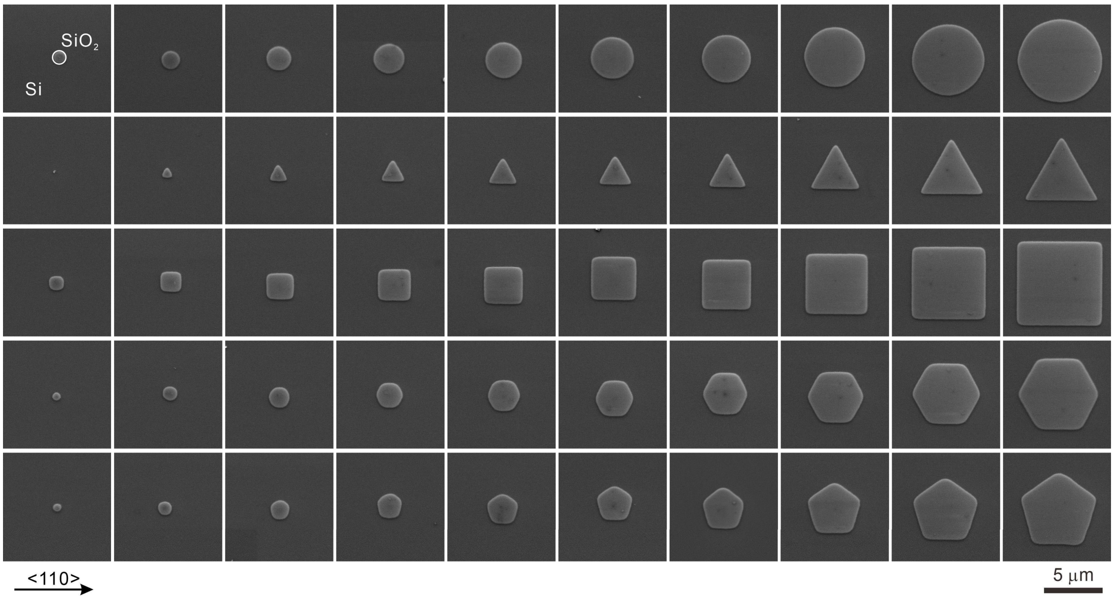

Fig. S3 SiO_2 masks patterned on Si (100) along Si [110]. The diameter of the mask varies from 0.4 μm to 7.5 μm.

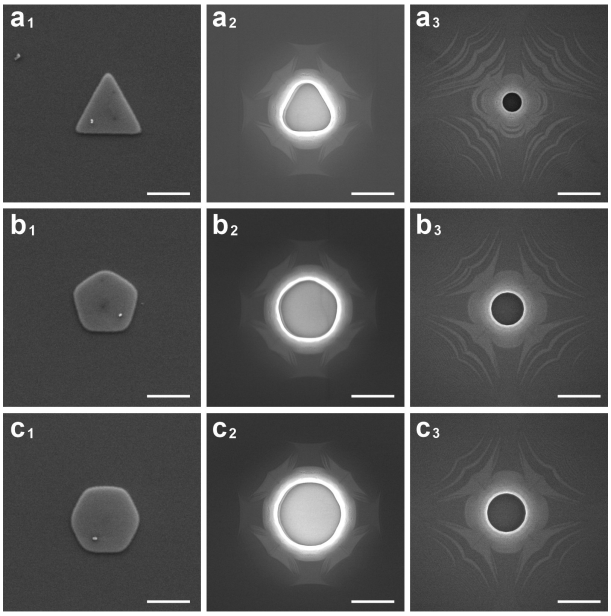

Fig. S4 Ge ELO on mask with different geometries. ELO first starts from the corner of masks and the growth velocity at concave corners is faster than at the edges. Anisotropic growth transitions to isotropic when the straight edges vanish, then the front grows inward with equal velocity. The scale bar is 2 μm.

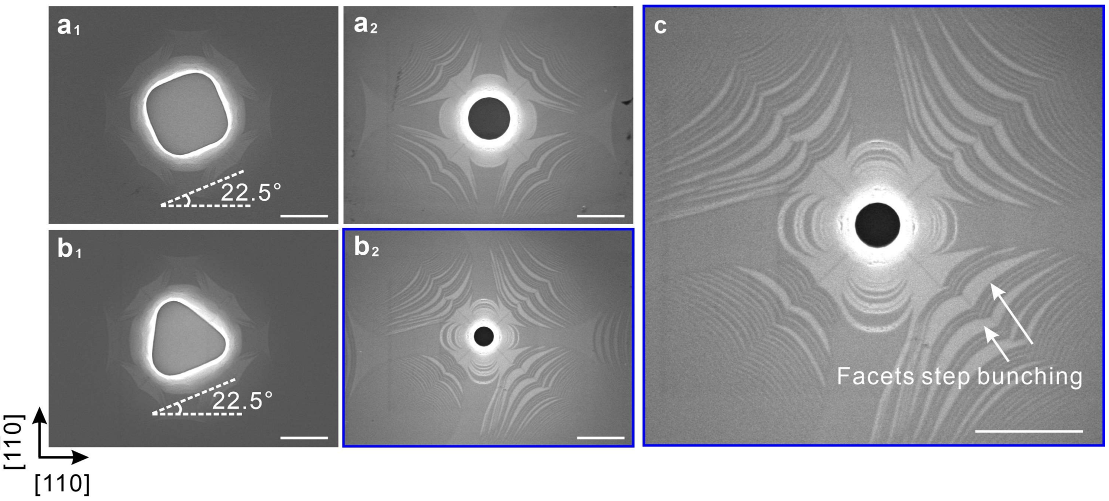

Fig. S5 a-b, Ge ELO on square and triangular mask with edges rotated 22.5° from Si [110], showing that the anisotropic-isotropic transition occurs irrespective of the angle between mask and Si [110]. **c**, enlarged view of (**b**$_2$) showing stripy contrast indicating that step bunches and surface facets are formed during growth. The scale bar is 2 μm.

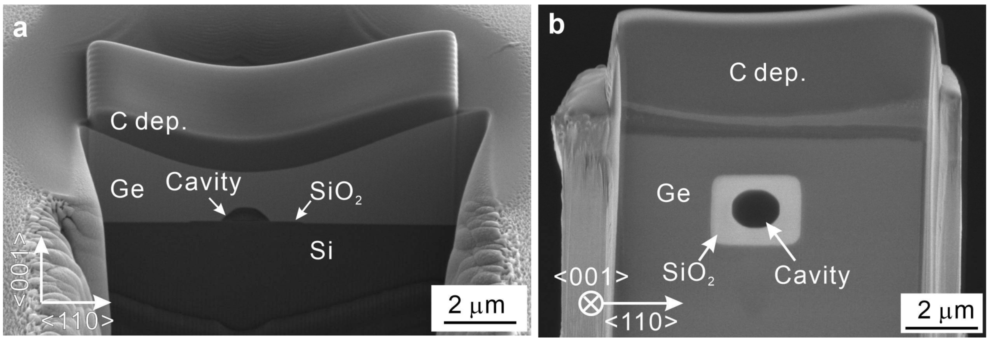

Fig. S6 (**a**) Cross-section SEM image and (**b**) Plan-view SEM image of a cavity formed after Ge full coalescence on mask. The samples are fabricated by FIB across the center of mask and at the Ge-Si interface, respectively. SEM images are taken at a tilt angle of 52°.

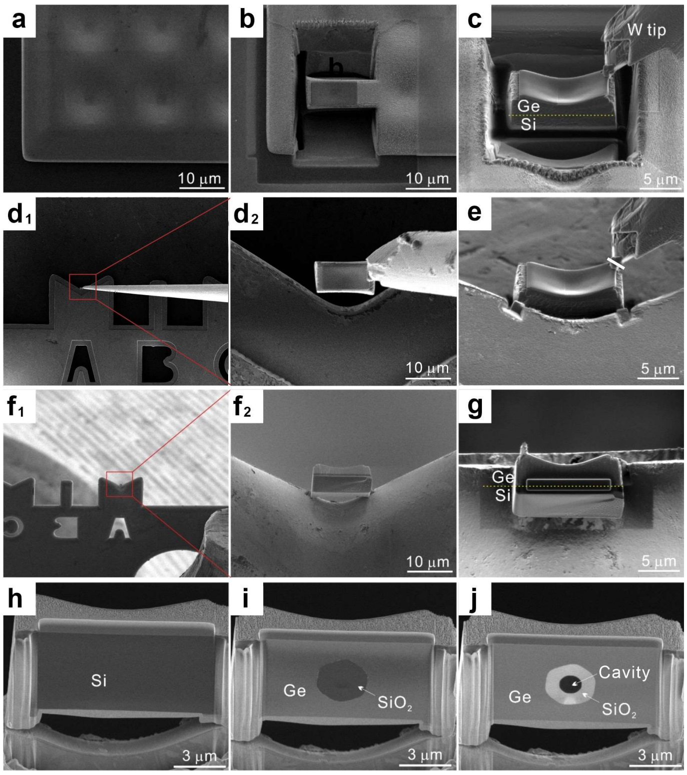

Fig. S7 FIB procedure for plan-view analysis of cavities on oxide. (**a-c**) Lift-out of a specimen containing Ge on oxide-patterned Si; (**d-e**): Top-view of specimen transfer to a grid; (**f-g**): rotated 90 ° for FIB milling; (**h-j**): Si substrate was milled step by step, and the cavity gradually appears at the Ge-oxide interface.

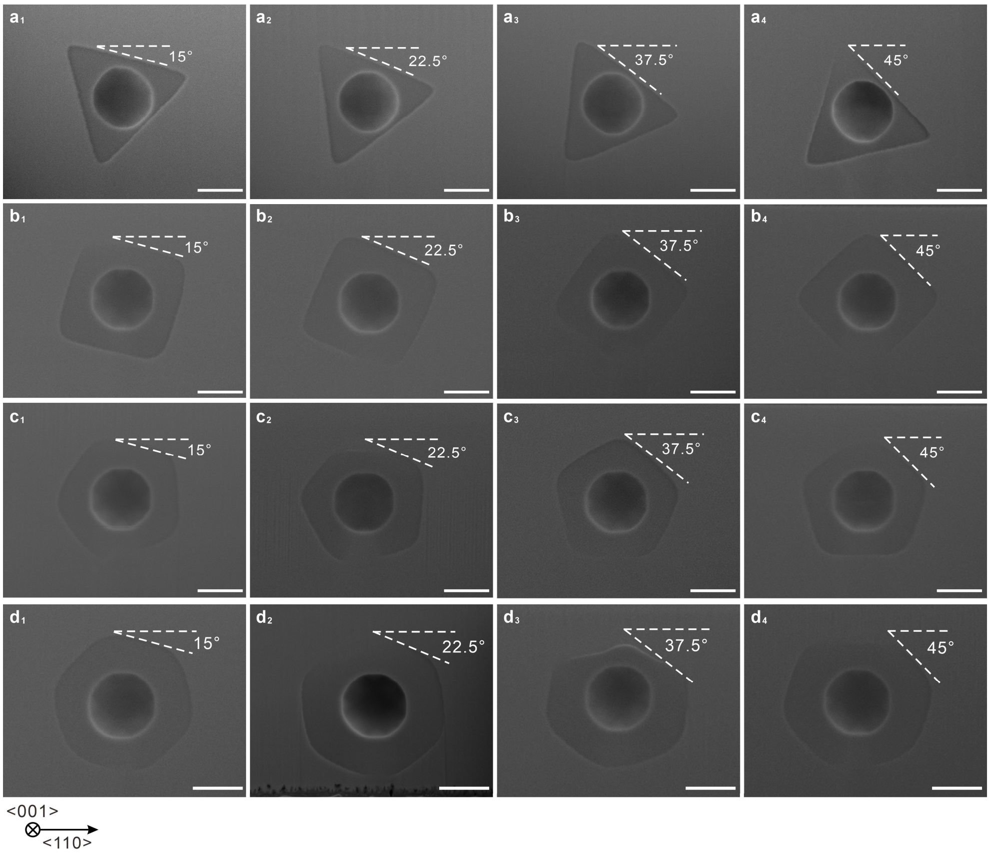

Fig. S8 Uniform cavities formed on triangular, quadrilateral, pentagonal, and hexagonal masks, irrespective of the angle between mask and Si [110].

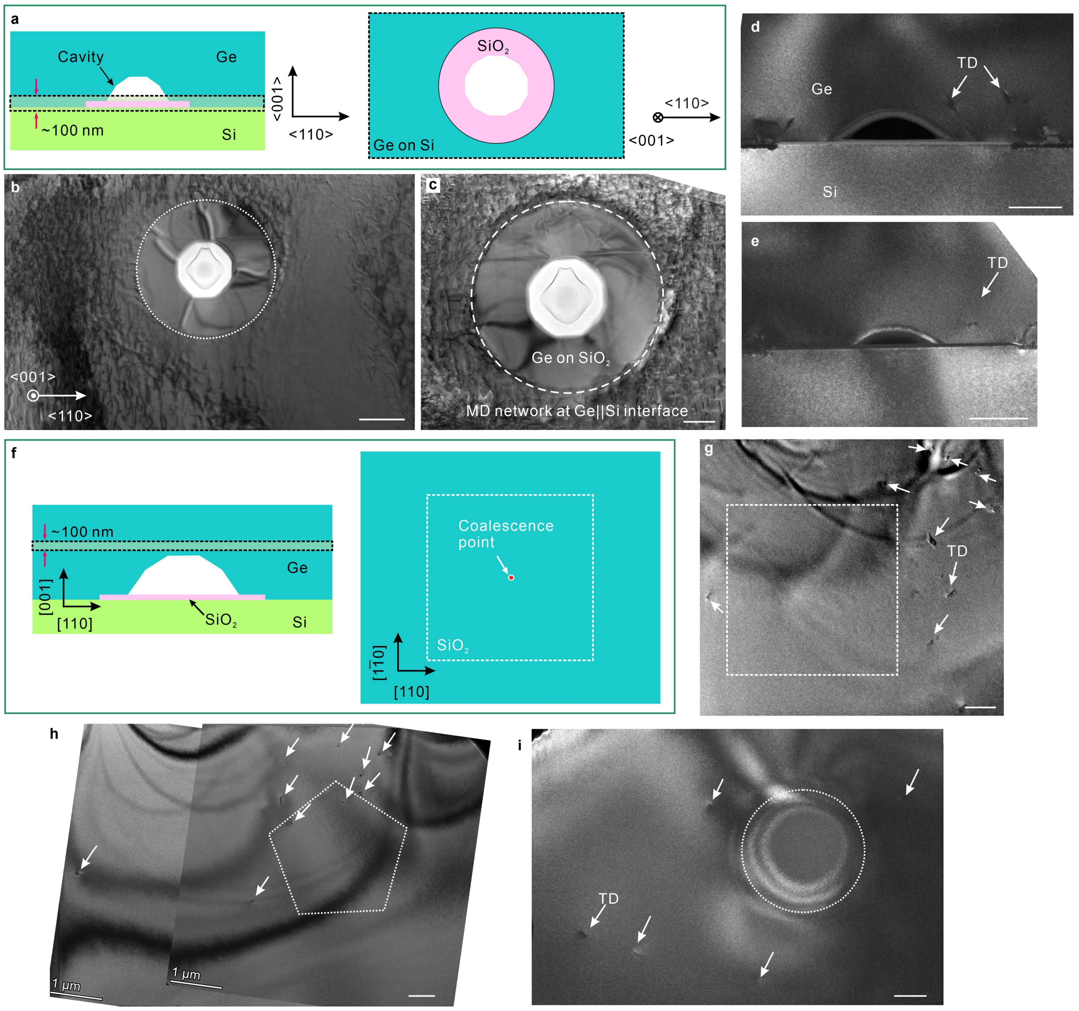

Fig. S9 a, Illustration of how a TEM specimen with thickness ~100 nm is fabricated by FIB to image the Ge/Si interface. **b**, Plan-view TEM image of the specimen at Ge/Si interface. **c**, is the higher magnification image, where the misfit dislocation network is observed at Ge/Si interface while dislocation-free Ge is found over the oxide. **d-e**, Dark field TEM image of cross-section view across the mask after full coalescence. **f**, Illustration of how a plan-view TEM lamella is fabricated above the cavity, with plane parallel to Si (001). The FIB milling process was from the Si to Ge, so that the position of the mask can be marked in the final TEM lamella. **g**, Plan-view TEM image of the Ge on top of mask at the coalescence point for the cavity, showing dislocation-free Ge at the area above the mask (dashed square) including the coalescence point, while threading dislocations are observed from the Ge grown on Si (white arrows). **h-i**, from the same position as illustrated in (**f**), Plan-view TEM lamella from a pentagon and round oxide pad were fabricated above the cavity, no threading dislocation was observed at the coalescence point (the center of dielectric mask). The scale bars are 500 nm.

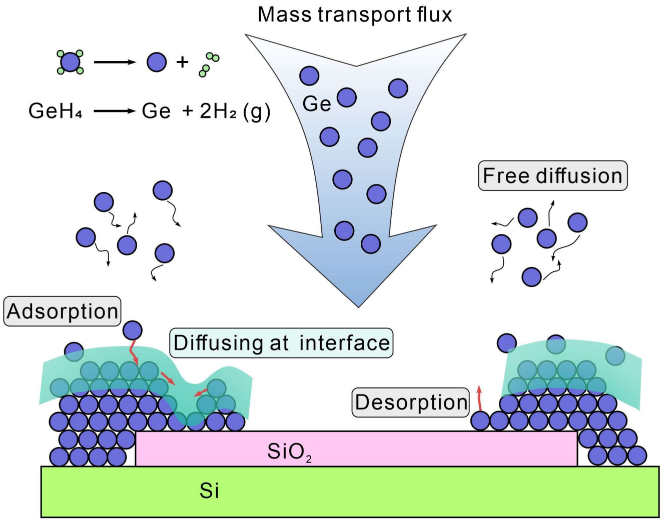

Fig. S10 A simplified illustration of concentration field in the growth model. Free diffusion in the gas atmosphere is considered. For the Ge adatoms at gas-crystal interface, there are three pathways for motion: adsorption, desorption, and diffusion. Additionally, Ge adatom diffusion at interface is mediated by curvature in the model.

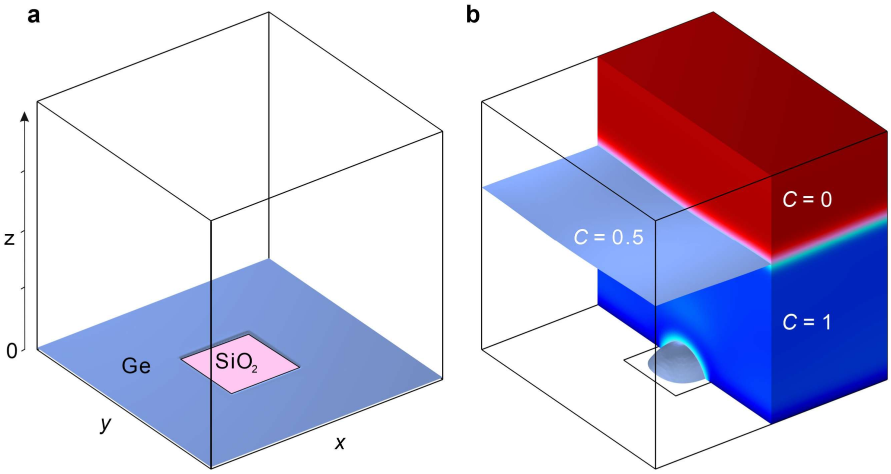

Fig. S11 Phase field model construction. a, Initial state of numerical simulation where a square mask was defined in the center of z = 0 plane of a cubic area. **b**, The Ge profile evolution is tracked through the order parameter C (volume fraction of solid Ge), with values 1 and 0 representing the solid phase (Ge crystal) and gas phase respectively, the interface between them being represented by the C=0.5 isosurface.

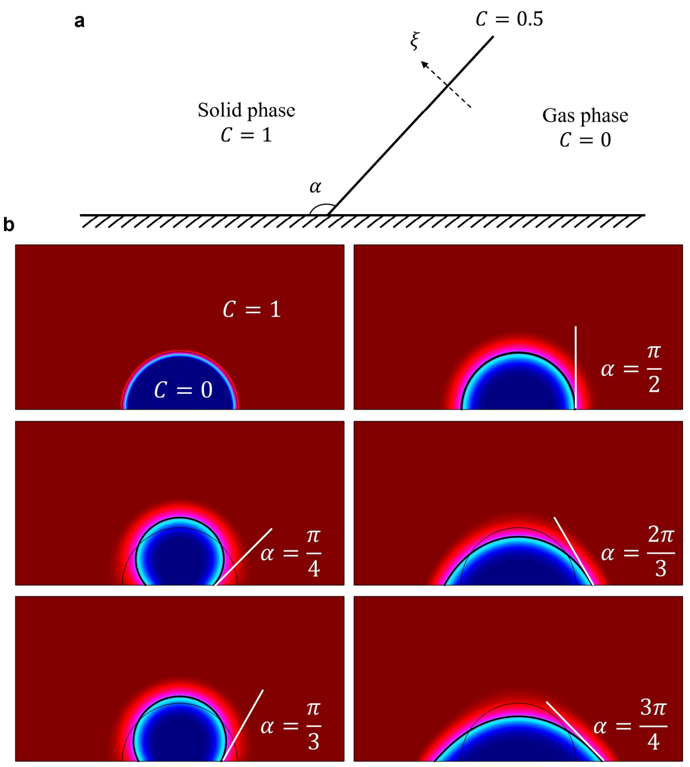

Fig. S12 **a**, Schematic of interface (a contour of C) at stasis. ξ is the local direction normal to the interface from gas phase to solid phase. α is the contact angle of solid Ge on mask. **b**, With different setting of the initial contact angle, the calculated morphology of the cavity inside the solid Ge at long time.

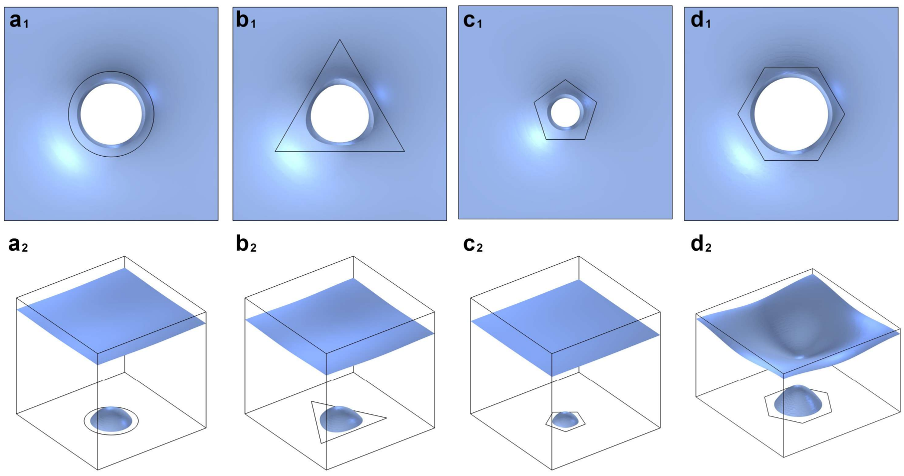

Fig. S13 Phase field simulation (PFS) of ELO process and cavity formation on mask with varied geometry. a_1-d_1: Top view of the simulation of growth front changing from anisotropic to isotropic and maintaining isotropic growth until it coalesced, irrespective of the mask shape (circle, triangle, pentagon and hexagon are shown). **a_2-d_2**: Cavities are formed at the center of mask after growth front coalescence, corresponding to **a_1-d_1**.

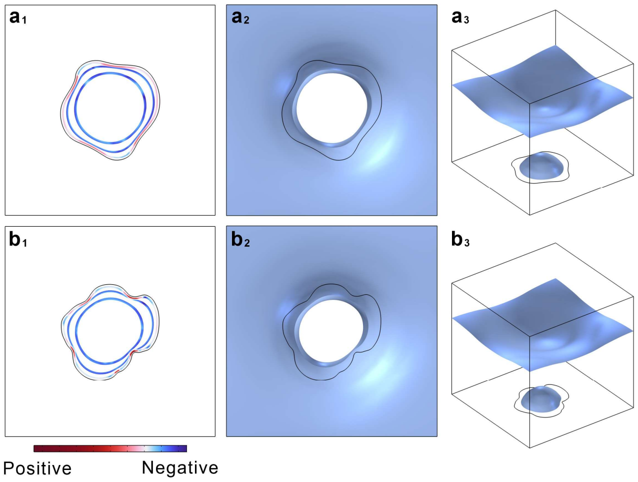

Fig. S14 Additional simulation of random masks. **a_1**, **b_1**: Illustrative scheme of the surface diffusion modeling, with the color map of the outline representing the curvature of mask while the curvature of the Ge growth front has the same value but opposite sign. **a_2**, **b_2**: Top view of the anisotropic to isotropic transition of ELO on the asymmetric masks. **a_3**, **b_3**: Side view of cavity formation after coalescence.

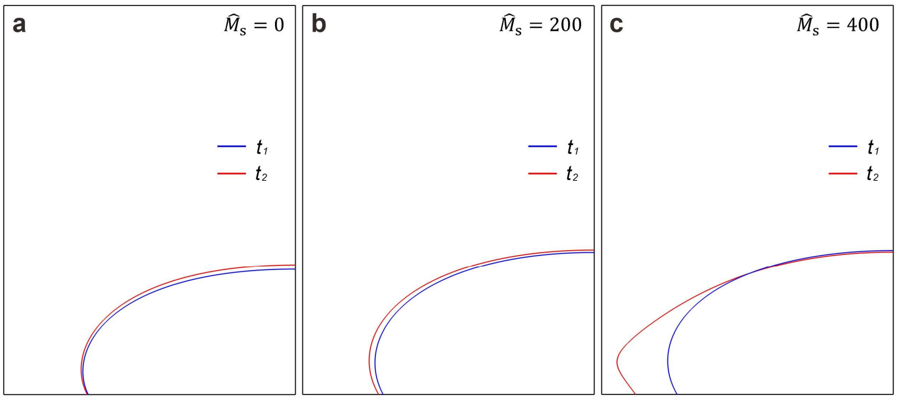

Fig. S15 Simulation of surface diffusion at different choices of the value of the mobility of $\widehat{M}_s$: **a**, no surface diffusion, $\widehat{M}_s = 0$; **b**, moderate surface diffusion, $\widehat{M}_s = 200$ and (**c**) surface diffusion chosen in studying kinetics $\widehat{M}_s = 400$. The Ge profile recedes from t_1 to t_2 in simulations where surface diffusion is set to be "strong" ($\widehat{M}_s = 400$) while recession is not observed when we set weaker surface diffusion $\widehat{M}_s = 200$ or zero surface diffusion $\widehat{M}_s = 0$.

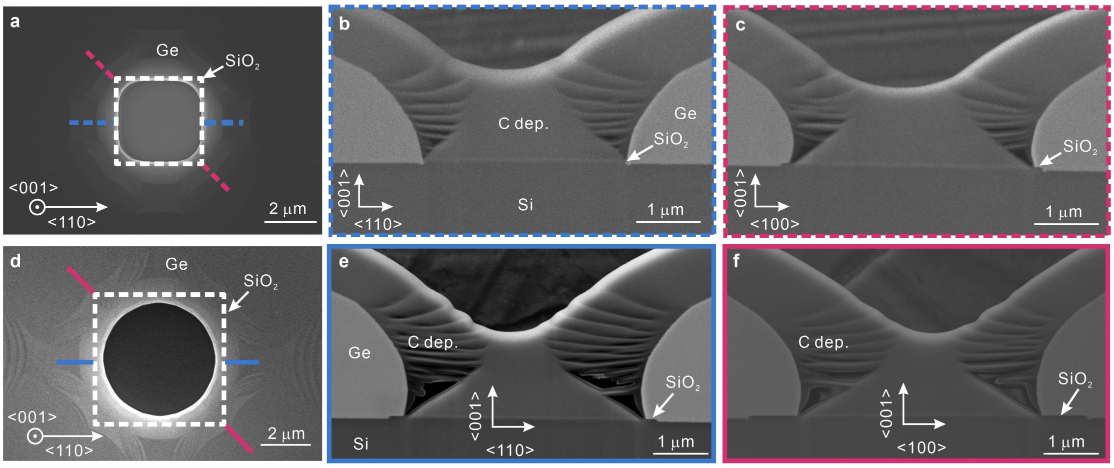

Fig. S16 Growth front morphology of anisotropic and isotropic ELO process. a, Top-view SEM image showing the accelerated rate of ELO at the corner of a quadrilateral mask. **b** and **c** are the cross-section views produced by FIB milling along Si[110] and Si[100] direction, respectively. C was deposited before FIB fabrication and the horizontal features are caused by the C_xH_y gas flow during deposition. The growth front at the mask edge is inhibited with contact angle smaller than 90° while the front at the mask corner moves forward with contact angle ~ 100°. **d-f** During isotropic-ELO process, the growth front is moving inward with uniform velocity and contact angle of ~ 100° along Si [110] and [100] directions.

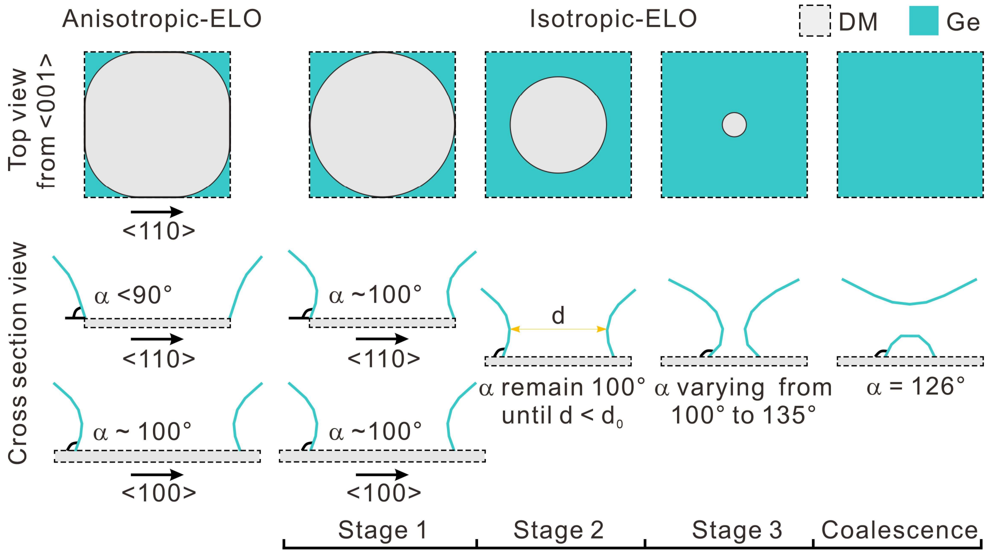

Fig. S17 Illustration of growth front morphology evolution under anisotropic- and isotropic-ELO processes. The contact angle α is smaller than 90° when the growth front is inhibited at the mask edge (orientation Si <110>), while α is ~100° when the growth front moves forward at the corner of the mask (Si <100>). The growth fronts become uniform when isotropic ELO begins; α remains ~100° until the diameter of the open cavity is smaller than d_0. The d_0 here denotes the critical diameter value of the transition zone where Ge adatom consumption cannot be supplied effectively thus causing accelerated growth of the front end and sluggish growth of the contact point. The contact angle then evolves from 100° to 135°, indicating a strong geometric shielding effect when the growth fronts are close enough. After full coalescence, the cavity is formed and the inner facets are reconfigured.

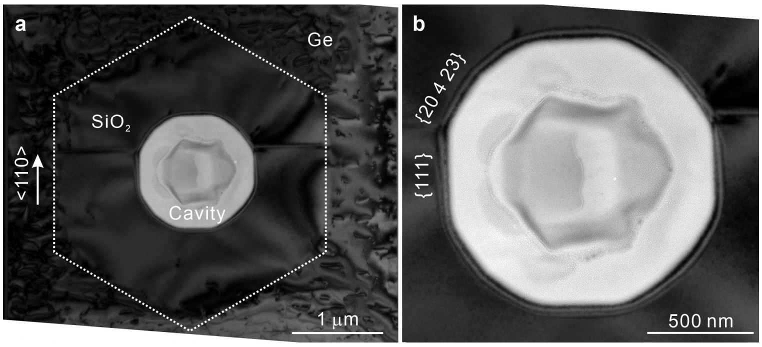

Fig. S18 a, Plan-view TEM image of Ge-SiO_2 interface showing cavity located at the center of a hexagonal mask. **b**, Enlarged image of cavity showing the cavity contact line with SiO_2 has 12 edges.

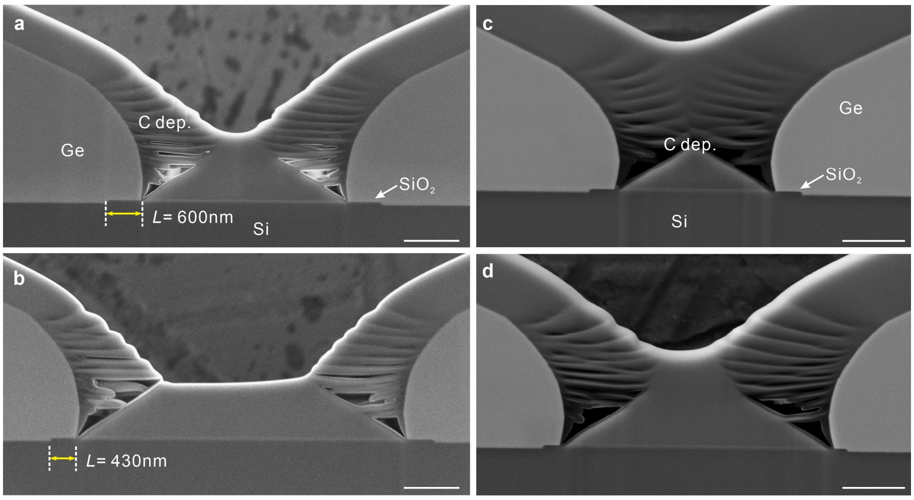

Fig. S19 a-b, Cross-section view of Ge ELO on round masks with different diameters. The mask diameters in **a** and **b** are 4.9 μm and 6.8 μm, respectively, and undergo the same growth condition and growth time. The epi-length (*L*) of each mask is shown on the images and indicate that smaller diameter masks undergo a faster growth rate with larger curvature. The contact angle of both is ~100°. **c-d**, Cross-section view of Ge ELO on square masks with different diameters. Viewed from Si [110] direction, the geometry of growth front is the same as **a** and **b**, indicating the contact angle between Ge and mask is determined by the interfacial energy between Ge and mask, and the curvature plays a minor effect at the early stages. The scale bars are 1 μm.

References

1. Cahn, J. W.; Hilliard, J. E. *The Journal of chemical physics* **1959,** 31, (3), 688-699.
2. Cahn, J. W.; Elliott, C. M.; Novick-Cohen, A. *European journal of applied mathematics* **1996,** , (3), 287-301.
3. Mullins, W. W. *J. Appl. Phys.* **1957,** 28, (3), 333-339.
4. Wixom, R. R.; Rieth, L. W.; Stringfellow, G. B. J. Crystal Growth 2004, 269, (2-4), 276-283
5. Yue, P. *Journal of Fluid Mechanics* **2020,** 899, A15.
6. Siem, E. J.; Carter, W. C. *J. Mater. Sci.* **2005,** 40, (12), 3107-3113.
7. Gai, Z.; Yang, W. S.; Zhao, R. G.; Sakurai, T. *Physical Review B* **1999,** 59, (23), 15230-15239.
8. Naffouti, M.; Backofen, R.; Salvalaglio, M.; Bottein, T.; Lodari, M.; Voigt, A.; David, T.; Benkouider, A.; Fraj, I.; Favre, L.; Ronda, A.; Berbezier, I.; Grosso, D.; Abbarchi, M.; Bollani, M. *Sci Adv* **2017,** 3, (11), eaao1472.
